# Establishment of a new human osteosarcoma cell line, UTOS-1: cytogenetic characterization by array comparative genomic hybridization

**DOI:** 10.1186/1756-9966-28-26

**Published:** 2009-02-25

**Authors:** Taketoshi Yasuda, Masahiko Kanamori, Shigeharu Nogami, Takeshi Hori, Takeshi Oya, Kayo Suzuki, Tomoatsu Kimura

**Affiliations:** 1 Department of Orthopaedic Surgery, Faculty of Medicine, University of Toyama, 2630 Sugitani, Toyama 930-0194, Japan; 2 Department of 2nd Pathology, Faculty of Medicine, University of Toyama, 2630 Sugitani, Toyama 930-0194, Japan

## Abstract

The cytogenetic characteristics of osteosarcoma (OS) remain controversial. The establishment of a new human OS cell line may improve the characterization. We report the establishment of a new human osteosarcoma cell line, UTOS-1, from a typical osteoblastic OS of an 18-year-old man. Cultured UTOS-1 cells are spindle-shaped, and have been maintained *in vitro *for over 50 passages in more than 2 years. Xenografted UTOS-1 cells exhibit features typical of OS, such as production of osteoid or immature bone matrix, and proliferation potency *in vivo*. UTOS-1 also exhibit morphological and immunohistochemical characteristics typical of osteoblastic OS. Chromosomal analysis by G-band show 73~85 chromosomes with complicated translocations. Array CGH show frequent gains at locus *DAB2 *at chromosome 5q13, *CCND2 *at 12p13, *MDM2 *at 12q14.3-q15, *FLI *and *TOP3A *at 17p11.2-p12 and *OCRL1 *at Xq25, and show frequent losses at *HTR1B *at 6q13, *D6S268 *at 6q16.3-q21, *SHGC17327 *at 18ptel, and *STK6 *at 20q13.2-q13.3. The UTOS-1 cell line may prove useful for biologic and molecular pathogenetic investigations of human OS.

## Introduction

Osteosarcoma (OS) is the most common malignant bone tumor in adolescents and young adults, and is characterized by proliferation of tumor cells which produce osteoid or immature bone matrix. Despite recent advances in multimodality treatment consisting of aggressive adjuvant chemotherapy and wide local excision, pulmonary metastasis occurs in approximately 40 to 50% of patients with OS and remains a major cause of fatal outcome [1-3].

There have been several reports describing xenotransplantation models of human OS [4-7], but characterization of human OS at the molecular cytogenetic level has been limited [8,9]. We describe the establishment and characterization of a new human OS cell line, designated as UTOS-1, derived from a conventional osteoblastic OS. In addition, we analyze chromosomal aberrations and DNA copy number changes in UTOS-1 by array comparative genomic hybridization (aCGH).

## Methods

### Source of Tumor Cells

**Figure 1:**
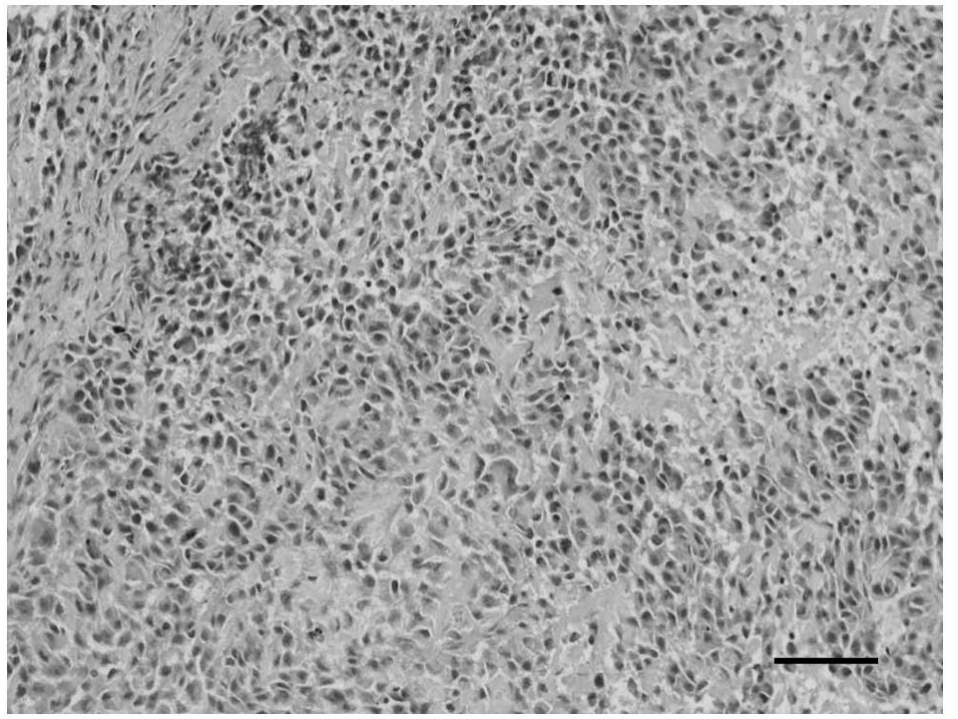
**Histologic appearance of the original tumor**. Spindle-shaped tumor cells with atypical nuclei have proliferated with formation of osteoid or immature bone matrix (H&E stain). Sclae bar: 100 μm.

An 18-year-old Japanese man noticed swelling and pain of the left shoulder for 2 months. Radiographs showed a periosteal reaction and an osteosclerotic change in the proximal metaphysis of the left humerus. An open biopsy of this humeral tumor confirmed that it was conventional osteoblastic OS (Figure 1). Immunohistochemically, most of the tumor cells were strongly positive for vimentin, alkaline phosphatase (ALP), osteopontin (OP), and osteocalcin (OC). Despite intensive chemotherapy, the patient died of lung metastasis 2 months after open biopsy. The present study was conducted after a human experimentation review by our ethics committee.

### Tumorigenicity in severe combined immunodeficiency (SCID) mice

To determine the tumorigenicity of the UTOS-1 cell line *in vivo*, 1 × 10^8 ^cells were washed, suspended in phosphate-buffered saline (PBS), and injected subcutaneously into the leg of 4-week-old male SCID mice (CB-17/Icrscid; Clea Japan Incorporation, Osaka, Japan). Also, tumor growth *in vivo *was measured by calculating tumor volume based on the measurement of 2 perpendicular diameters using a caliper [10]. The volume was estimated using the following formula: 0.5 × L × (S)^2^, where L and S are the largest and smallest perpendicular tumor diameters, respectively.

### Establishment of the tumor cell line

Tumor cells were seeded in a 25 cm^2 ^plastic flask (35–3109; Falcon, Franklin Lakes, NJ, USA) [11]. These cells were cultured in RPMI 1640 (MP Biomedicals, Solon, OH, USA), supplemented with 100 mg/ml streptomycin (Meiji Seika, Tokyo, Japan), 100 U/ml penicillin (Meiji Seika) and 10% fetal bovine serum (FBS; Funakoshi, Tokyo, Japan), at 37°C in a humidified atmosphere of 5% CO_2 _and 95% air. The medium was replaced once per week. When semiconfluent layers were obtained, the cells were dispersed with Ca^2+^- and Mg^2+^-free PBS containing 0.1% trypsin and 0.02% EDTA solution, and were then seeded in new flasks for passage. The configuration of tumor cells was almost equalized after the 3rd generation. These procedures were serially performed until the UTOS-1 cell line was established.

### Cell growth in vitro

To determine the doubling time, UTOS-1 cells were seeded in each well of 96-well dishes (Corning Costar, Tokyo, Japan) with fresh medium containing 100 μl of RPMI 1640 with 10% FBS. Cell growth was measured using the 3-(4,5-dimethylthiazol-2-yl)-2,5-diphenyltetrazolium bromide (MTT) assay (Cell Counting Kit-8, Dojindo, Tokyo, Japan) [12]. A volume of 10 μl of MTT was added to each well, followed by mixing. Plates were incubated for 3 hours at 37°C in a humidified atmosphere of 5% CO_2 _and 95% air. Formazan levels, which correspond to the number of viable cells, were quantified using a microplate reader (model 450; Bio-Rad Laboratories, Hercules, CA, USA) at a wavelength of 450 nm. The absorbance of each well was evaluated at 6, 12, 24, 48, 72, 96 and 120 hours after seeding. Triplicate wells were used for each observation.

### Immunohistochemistry

Cells were cultured in chamber slides (Lab-Tek; Nalge Nunc International, Naperville, IL, USA). For the detection of mesenchymal phenotype, we used 3 monoclonal antibodies: anti-AE1/AE3, anti-keratin mix, and anti-vimentin. Also, to assess osteoblastic differentiation, we used 2 monoclonal antibodies: anti-OP and anti-OC. ALP activity of UTOS-1 cells was estimated using a modified version of a cytochemical method described elsewhere [13], with naphthol AS-MX phosphate-fast blue RR staining (ALP staining kit; Muto Pure Chemicals Corporation, Tokyo, Japan).

Cells grown in chamber slides were washed in PBS, fixed in 4% paraformaldehyde for 15 minutes at room temperature, and then fixed in methanol for 20 minutes at -20°C. The cells were incubated with each of the primary antibodies for 24 hours at 4°C. Immunoreaction products were detected using DAKO envision (DAKO Sytomation, Carpinteria, CA, USA), and were visualized after adding diaminobenzidine (DAB; DAKO) as the chromogen.

### RNA extraction and reverse-transcription polymerase chain reaction (RT-PCR)

Expression of osteoblastic differentiation markers was assessed using RT-PCR. UTOS-1 cells were grown to confluence, and total cellular RNA was isolated using a TRIzol^® ^Reagent (Invitrogen, San Diego, CA, USA). Total RNA was used as a template for cDNA synthesis using the SuperScript First-strand Synthesis System (Invitrogen). PCR was performed to assess expression of ALP, OP and OC. The oligonucleotide primer sequences and PCR conditions for ALP, OP and OC are shown in Table 1. Amplified products were analyzed by 2% agarose gel (Cambrex Bio Science Rockland Incorporation, Rockland, ME, USA) electrophoresis and ethidium bromide staining (Invitrogen). For comparison, Saos-2 [7], which is one of the most popular OS cell lines, was used as a positive control.

**Table 1:** The oligonucleotide primer sequences and PCR conditions for ALP, OP, and OC in this study.

Molecule	Primers (5' to 3')	Strand	Size (bp)	Conditions (temperature, cycle number)
ALP	ACGTGGCTAAGAATGTCATC CTGGTAGGCGATGTCCTTA	+ --	475	55°C 35 cycles
OP	CCAAGTAAGTCCAACGAAAG GGTGATGTCCTCGTCTGTA	+ --	347	58°C 45 cycles
OC	ATGAGAGCCCTCACACTCCTC GCCGTAGAAGCGCCGATAGGC	+ --	294	59°C 45 cycles
GAPDH	GAAGGTGAAGGTCGGAGTCA GAAGATGGTGATGGGATTTC	+ --	226	55°C 35 cycle

Abbreviations: ALP, alkaline phosphatase; OP, osteopontin; OC, osteocalcin; GAPDH, glyceraldehyde-3-phosphate dehydrogenase.

### Cytogenetic analysis

For cytogenetic analysis, preparations of metaphase chromosomes from UTOS-1 cells at passage 15 were obtained, and were banded with Giemsa-trypsin [14]. Karyotypes were described using the short version of the International System for Human Cytogenetic Nomenclature [15].

### DNA extraction and array CGH

Genomic DNA was extracted from UTOS-1 cells at passage 15. The CGH procedure used was similar to published standard protocols [16]. Genomic DNA was isolated from tumor samples using standard procedures including proteinase K digestion and phenol-chloroform extraction.

Array CGH was performed using the GenoSensor Array 300 system, following the manufacturer's instructions (Vysis, Downers Grove, IL, USA). This array contains the 287 chromosomal regions that are commonly altered in human cancer, such as telomeres, regions involved in microdeletions, oncogenes, and tumor suppressor genes. Tumor DNA (100 ng) was labeled by random priming with fluorolink cy3-dUTP, and normal reference (control) DNA was labeled using the same method with cy5-dUTP. The tumor and control DNAs were then mixed with Cot-1 DNA (GIBCO-BRL, Gaithersburg, MD, USA), precipitated, and resuspended in microarray hybridization buffer containing 50% formamide. The hybridization solution was heated to 80°C for 10 minutes to denature the DNA, and was then incubated for 1 hour at 37°C. Hybridization was performed for 72 hours in a moist chamber, followed by a post-hybridization wash in 50% formamide/2 × SCC at 45°C. Slides were mounted in phosphate buffer containing 4',6-diamidino-2-phenylindole (DAPI; Sigma, St. Louis, MO, USA). Fluorescence intensity images were obtained using the GenoSensor Reader System (Vysis) according to the manufacturer's instructions. For each spot, the total intensity of each of the 2 dyes and the ratio of their intensities were automatically calculated. The diagnostic cut-off levels representing gains and losses were set at 1.2 (upper threshold) and 0.8 (lower threshold). This assay was performed in triplicate, and common aberrations were considered to be meaningful aberrations.

## Results

### Tumor growth in vivo

**Figure 2:**
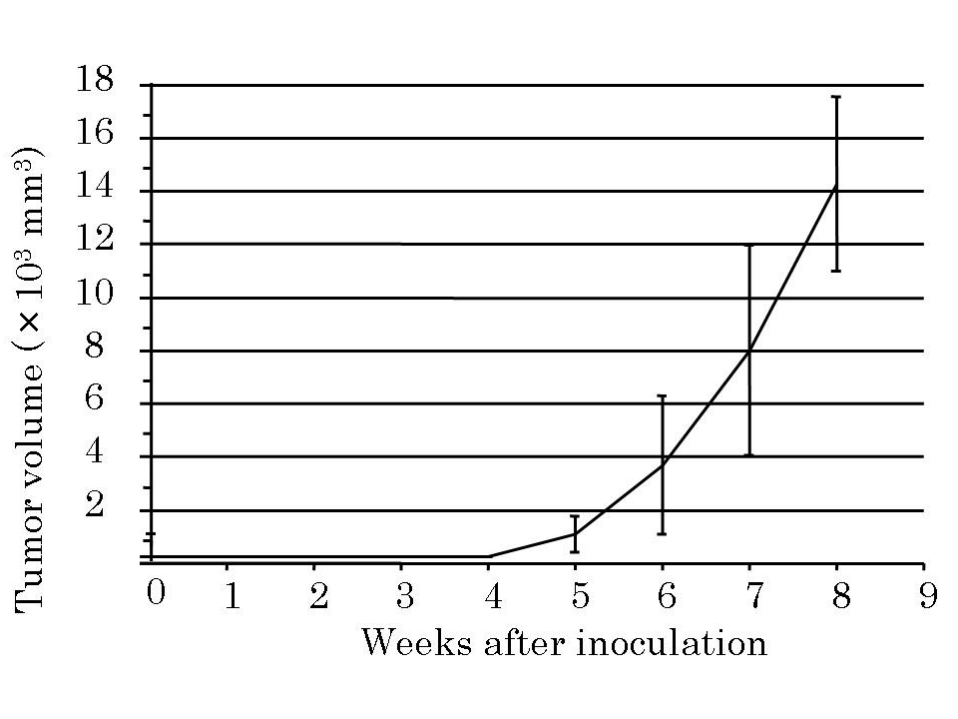
**Tumor volume in SCID mice**. Tumor volume in logarithmic growth phase, ~5 weeks after inoculation. Values are expressed as the mean ± standard deviation of triplicate cultures.

**Figure 3:**
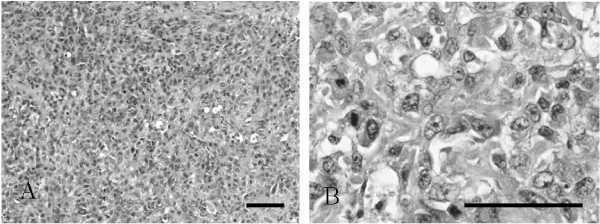
**Histologic appearance of xenografted tumor in SCID mice**. **A**. Xenografted tumor showing features typical of osteoblastic osteosarcoma with atypical spindle-shaped cells (H&E stain). Scale bar: 100 μm. **B**. The proliferation of atypical tumor cells with osteoid formation is shown. Xenografted tumor cells resemble original tumor cells. Scale bar: 50 μm.

Approximately 5 weeks after implantation, all SCID mice had palpable elastic hard nodules with a volume of about 1000 mm^3 ^(Figure 2). The tumor volume was about 4000 mm^3 ^at 6 weeks after implantation, and was > 10,000 mm^3 ^at 8 weeks after implantation. The cut surfaces of these tumors were solid and white-gray with small necrotic foci. Histopathologically, the tumors contained primarily atypical tumor cells, and exhibited formation of osteoid or immature bone matrix, which is similar in characteristics to the original tumor (Figure 3).

### Cell growth and morphological findings in vitro

**Figure 4:**
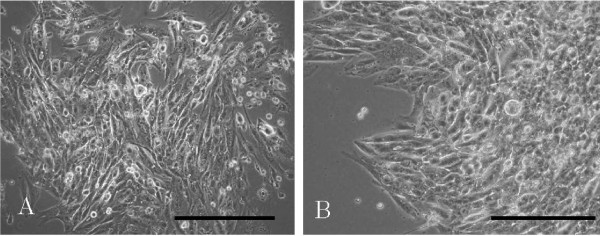
**Morphology under phase-contrast microscopy**. **A**. In primary culture, spindle-shaped tumor cells reach subconfluence with some piled-up foci of cells. Scale bar: 100 μm. **B**. At passage 6, the tumor cells begin to grow rapidly. The configuration of tumor cells is equalized after the 6th generation. Scale bar: 100 μm.

UTOS-1 cells were spindle-shaped, contained several nucleoli, and formed clumps. Two weeks after initial cultivation in primary culture, the tumor cells reached subconfluence with some piled-up foci of cells (Figure 4A). After the cells were serially subcultured for about 3 months, they began to grow rapidly at passage 6 (Figure 4B).

**Figure 5:**
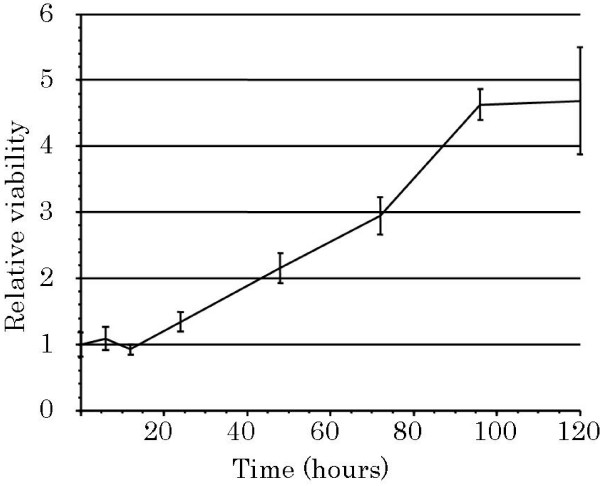
**Tumor cell growth in vitro**. UTOS-1 cells begin to grow ~24 hours after inoculation. The population-doubling time of the cells is 40 hours. Values are expressed as the mean ± standard deviation of triplicate cultures.

This new cell line has been maintained *in vitro *for more than 50 passages over more than 2 years. In the exponential phase of cell growth, the population-doubling time was 40 hours (Figure 5).

### Immunohistochemical and cytochemical findings

**Figure 6:**
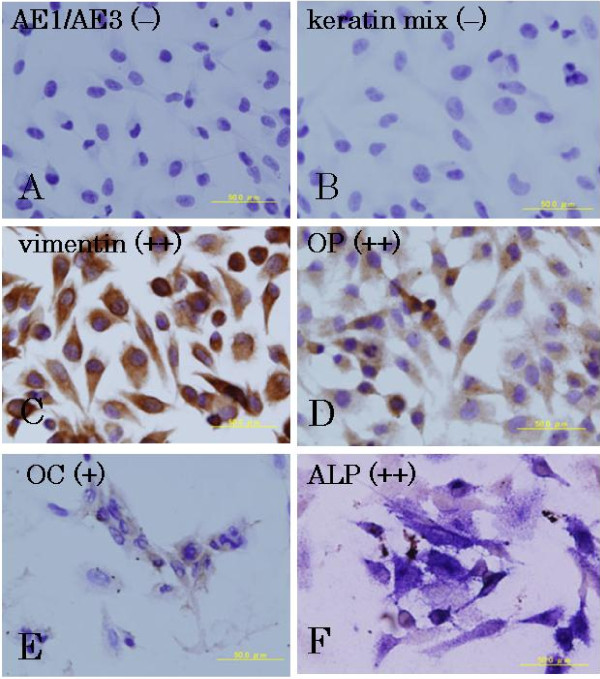
**Immunohistochemical findings**. **A, B. **UTOS-1 cells are negative for AE1/AE3 and keratin mix. **C, D, E. **Most UTOS-1 cells are positive for vimentin, OP, and OC. **F**. Staining for ALP was performed using a modified cytochemical method. ALP activity is visible as blue staining. UTOS-1 cells are strongly positive for ALP.

All UTOS-1 cells were negative for AE1/AE3 and keratin mix. Most UTOS-1 cells were positive for vimentin. All UTOS-1 cells were positive for OP, OC and ALP (Figure 6).

### RT-PCR

**Figure 7:**
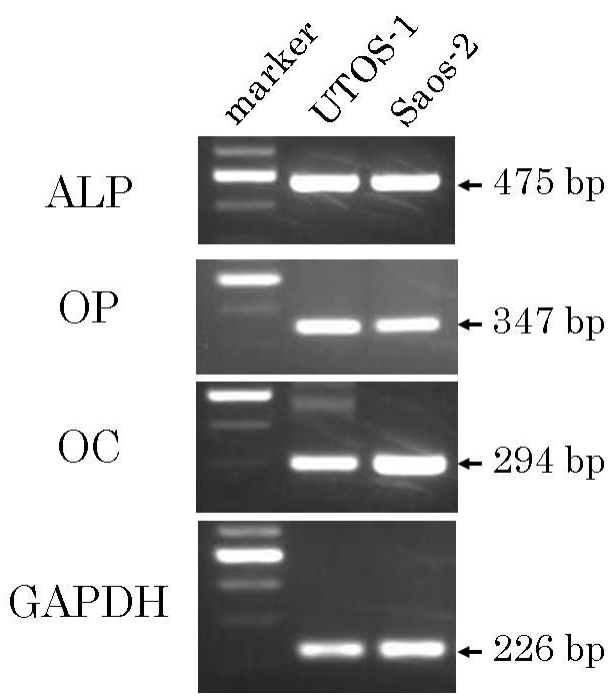
**Osteoblast marker expression in UTOS-1 cells**. The expression of several osteoblast markers, including ALP, OP and OC, is shown. Saos-2, which is one of the most popular OS cell lines, is used as a positive control for osteoblastic markers in UTOS-1 cells. These cells express ALP, OP and OC, which is similar to Saoa-2.

UTOS-1 cells expressed ALP, OP and OC, which is similar to the results for Saos-2 (Figure 7).

### Cytogenetic findings

**Figure 8:**
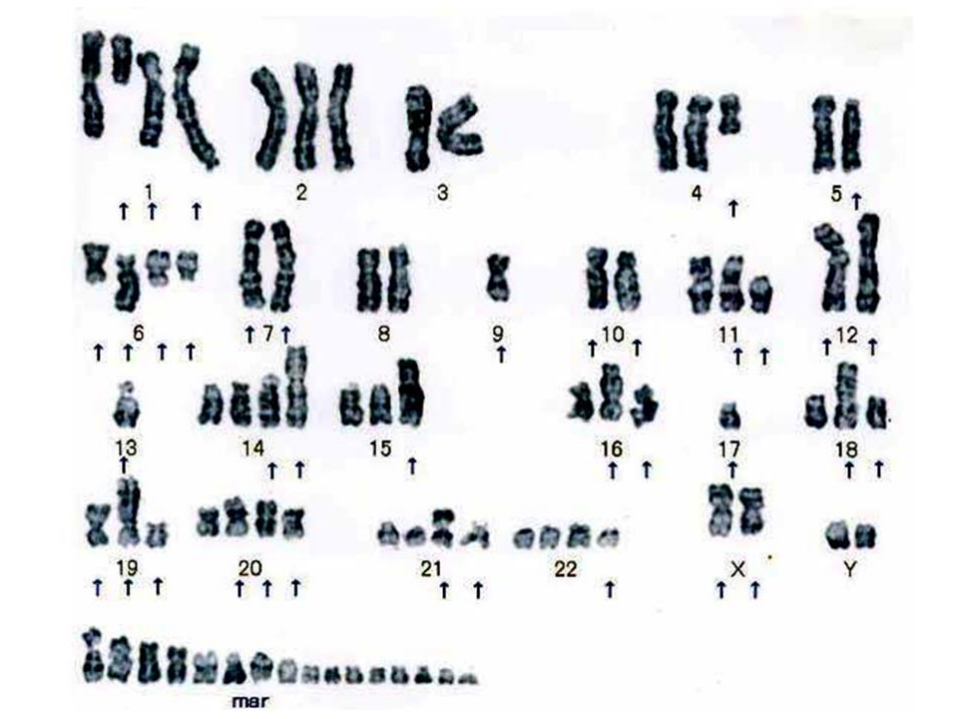
**A representative G-banded karyotype of a UTOS-1 cell**. Arrows show the abnormal chromosomes.

A representative karyotype is shown in Figure 8. 50 UTOS-1 cells exhibited a complex karyotype. The karyotypes of UTOS-1 cells at passage 15 were similar to those of the original tumor. The composite karyotypes were as follows: 73~85, Y, -X [7],+Y[10],add(X)(q11)[9],add(X)(q11)[2],+1[8],del(1)(q11)[9], der(1)add(1)(p11)add(1)(q42)[9],der(1)add(1)(p22)add(1)(q32)[2],der(1)add(1)(p32)add(1)(q42)[6],-3[10],-4[3],add(4)(q11)[9],-5 [4],del(5)(p13)[9], +add(6)(q11)[3],der(6)del(6)(p21)add(6)(q2?)[10],der(6)del(6)(p24)add(6)(q13) ×2,[10]-7[10],add(7)(p22)[6],der(7)t(7;7)(p22;q22)[10],+8[3],-9[10],-9[8],add(9)(q22)[9],-10[10],add(10)(p11)[4],add(10)(q26)[7], der(10)add(10)(p11)add(10)(q26)[3],add(11)(p11)[9],add(11)(p11)[4], del(11)(p11)[6],-12[5],der(12)(q21)[6],der(12)add(12)(p11)add(12)(q24)[10], der(12)add(12)add(12)[7],-13[10],+14[2], add(14)(p11)[10],add(14)[2], add(14)(p11)[8],-15[7],add(15)(p11)[5],add(15)(p11)[4],+16[5], add(16)(p11)[3],add(16)(q24)[2],add(16)(p11)[10],add(16)[4],-17[10],-17[8],add(17)(q24)[3],?del(17)(p11)[3],-18[5],add(18)(p11)[9], add(18)(q21)[5],+19[5],add(19)(p11)[9],add(19)(q13)[8],del(19)(p13)[9],+20[7],add(20)(p11)[7],add(20)(p13)[8],add(20)(q11)[4],+21[8],+21[4],add(21)(p11)[10],add(21)(p11)[4],add(21)(q22)[7],+22[10],+22[7],+22[3],del(22)(q13)[10],del(22)[9],+10~18 mar.

### Array CGH

**Figure 9:**
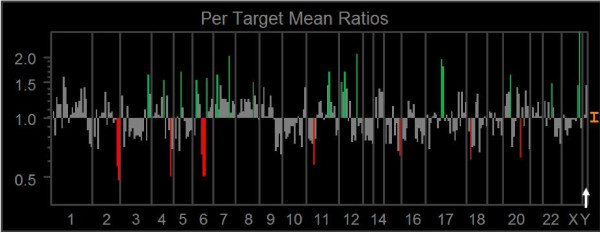
**Genetic instability analyzed by aCGH**. The line in the middle (gray) is the baseline ratio (1.0); The upper (red) and lower (green) bars in each frame indicate losses and gains, respectively. The arrow shows the axes of X and Y chromosomes.

Significant gains of DNA sequences were observed for locus *DAB2 *at chromosome 5q13, *CCND2 *at 12p13, *MDM2 *at 12q14.3-q15, *FLI, TOP3A *at 17p11.2-p12, and *OCRL1 *at Xq25. Significant losses of DNA sequences were observed for *HTR1B *at 6q13, *D6S268 *at 6q16.3-q21, *SHGC17327 *at 18ptel, and *STK6 *at 20q13.2-q13.3. The representative aCGH profile is shown in Figure 9.

## Discussion

There have been several reports describing xenotransplantation models of human OS [4-7]. In the present study, the parent tumor, the cultured tumor cells, and the xenografted tumor exhibited features typical of OS, as reported previously [15,17]. Cultured UTOS-1 cells have a spindle shape with several nucleoli, which is similar to the original tumor cells. Biochemical characteristics of UTOS-1, such as cell growth rate and osteoblastic activity, have not changed during the 2 years that they have been maintained. Immunohistochemically, the UTOS-1 cells remain positive for ALP, OP and OC. After implantation from cell culture into SCID mice, UTOS-1 cells grew *in vivo*, producing osteoid resembling that of the original tumor. Abundant osteoid tissue formed in the xenografted tumors and reimplanted tumors. These findings suggest that UTOS-1 cells have an osteoblastic phenotype and retain the characteristics of the original tumor. The population-doubling time of UTOS-1 cells *in vitro *is 40 hours, which is similar to that of other OS cell lines [4,6,18].

Several reports indicate that OS cells have karyotypes with multiple numerical rearrangements and complex structural rearrangements [9,19-21]. Together, the results of several cytogenetic surveys indicate that OS cells frequently have structural alterations at chromosome bands 1p11-13, 1q11-12, 1q21-22, 11p15, 12p13, 17p11-3, 19q13, and 22q11-13, and frequently have the numerical chromosome abnormalities +1, -9, -10, -13, and -17. In UTOS-1 cells, the clonal chromosomal abnormalities that were detected were triploidies. The chromosomal rearrangements that were observed in UTOS-1 involved chromosomes 1q11-12, 11p15, 19q13, and 22q11-13. The numerical chromosome abnormalities that were observed in UTOS-1 included +1, -9, -10, -13, and -17. These findings are similar to studies of other OS cell lines [8].

Metaphase CGH studies of OS have identified frequent gains at chromosome bands 1p32, 1q21, 5p13, 6p12, 8q24, 8cen-q13, 17p11.2, and Xp21, and frequent losses at bands 6q16, 10p12pter, and 10q22-q26 [22,23]. Recent metaphase CGH studies of OS have focused on amplifications at chromosomes 8q, 6p, and 17p [22,24]. Advances in mapping resolution of microarray CGH [25,26] have greatly improved its resolving power, such that it now provides greater detail than metaphase CGH regarding the complexity and exact location of genomic rearrangements leading to copy number imbalances.

In the present study, chromosome 12 showed several distinct regions of focal amplification, occurring at gains of *CCND2 *at 12p13 12q13 and *MDM2 *at 12q14.3-q15. Previous CGH studies of OS have revealed abnormalities of chromosome 12, including gains at bands 12p12-p13 [24], 12q12-q13 [27], and 12q13-q14 [28]. Expression of the *CCND2 *gene, which is located at chromosome 12p13, has been observed in various malignancies, including prostate cancer and breast cancer [29-31]. *CCND2 *encodes a protein belonging to the cyclin family of proteins that regulate cyclin-dependent kinase (CDK) kinases [32]. CDK activity controls the cell cycle G1/S transition by regulating phosphorylation of the tumor suppressor protein Rb [33]. These facts suggest that *CCND2 *controls proliferation of UTOS-1 tumor cells.

Some studies indicate that 14 to 27% of OS tumors have abnormal *MDM2 *expression [34,35]. *MDM2 *is a target gene of the transcription factor tumor protein p53 [36]. The encoded protein is a nuclear phosphoprotein that binds and inhibits transactivation by tumor protein p53, as part of an autoregulatory negative feedback loop [37,38]. Overexpression of *MDM2 *gene can result in excessive inactivation of tumor protein p53, diminishing its tumor suppressor function. These findings suggest the possible involvement of the *p53 *tumor suppressor gene, which is associated with development of OS in UTOS-1 cells.

The gain of chromosome band at 17p11.2-p12 has been observed in approximately 13 to 29% of high-grade OS [24,39,40]. In UTOS-1 cells, gain of the genes *FLI *and *TOP3A *at chromosome 17p11.2-p12 has been observed. These findings suggest that multiple gains, including *FLI, TOP3 *or other genes close to these candidate oncogenes, are present at chromosome 17p11.2-p12 and contribute to OS tumorigenesis [41]. Recent studies indicate that overexpression of 17p11.2-p12 is associated with p53 degradation [42-44].

In a study of OS using a cDNA array, Squire et al. observed amplification of the genes *MYC*, *GAS7*, and *PM1 *in OS cells [45]. Other reports indicate that losses of chromosome bands 6q16 and 6q21-q22 occur in high-grade OS [46]. These findings and those of the present study suggest that gene losses on chromosome 6q, including *HTR1B *and *D6S268*, contribute to OS tumorigenesis.

One of the most remarkable breakpoint clusters that have been found in OS tumors was detected on chromosome 20 by spectral karyotyping (SKY) analysis [47]. Chromosome 20 is one of the smaller chromosomes, suggesting that it is particularly vulnerable to structural rearrangement. However, there is little evidence that chromosome 20 is frequently involved in chromosomal imbalances [26,28]. In the present study, the only loss that involved chromosome 20 occurred at band 20q13.2-q13.3. Many chromosomal changes have been observed in CGH studies of high-grade OS [46]. Reports indicate that the genes involved in OS tumorigenesis include *DAB2 *(at chromosome 5q13), *OCRL1 *(at Xq25), and *SHGC17327 *(at 18ptel). However, many of these genes were not previously known to be associated with OS tumorigenesis.

In conclusion, we have isolated and characterized a new permanent human cell line, UTOS-1, established from an osteoblastic OS. This cell line retains the morphology, osteoblastic activities and cytogenetic characteristics of the original tumor *in vitro*. The UTOS-1 cell line is useful for biologic and molecular pathogenetic studies of human OS.

## Competing interests

The authors declare that they have no competing interests.

## Authors' contributions

Authors have made substantial contributions to conception and design MK and TY acquisition of data. SN, TH, TO and KS analysis, interpretation of data, organizing study. TY and supervision of research group TK
